# The Effects of Low Concentrations of Pravastatin on Placental Cells

**DOI:** 10.1007/s43032-024-01611-x

**Published:** 2024-06-05

**Authors:** Masako Kanda, Keiichi Kumasawa, Kazunari Nemoto, Risa Miyatake, Kei Inaba, Seisuke Sayama, Takahiro Seyama, Takayuki Iriyama, Takeshi Nagamatsu, Tomoyuki Fujii, Yasushi Hirota, Yutaka Osuga, Tadashi Kimura

**Affiliations:** 1 Department of Obstetrics and Gynecology, Graduate School of Medicine, Osaka University, Suita, Osaka, Japan; 2 Department of Obstetrics and Gynecology, Faculty of Medicine, the University of Tokyo, Bunkyo-ku, Tokyo, 113-8655 Japan; 3 Department of Obstetrics and Gynecology, Tokyo Metropolitan Toshima Hospital of the Tokyo Metropolitan Hospital Organization, Itabashi-ku, Tokyo, Japan; 4 Department of Obstetrics and Gynecology, Tokyo-Kita Medical Center, Kita-ku, Tokyo, Japan; 5 Department of Obstetrics and Gynecology, International University of Health and Welfare Narita Hospital, Chiba, Japan; 6 Department of Obstetrics and Gynecology, Sanno Hospital, Minato-ku, Tokyo, Japan

**Keywords:** Statin, Preeclampsia, Low concentration, Low-dose, Prevention, In vitro

## Abstract

Pravastatin is a promising medication to treat preeclampsia. However, the appropriate dose of pravastatin for managing preeclampsia has not been established. In this in vitro study, we examined the effects of low concentrations of pravastatin (0.01 to 10 µM) under hypoxic conditions on two types of placental cells and found that pravastatin decreased sFlt-1 levels up to 34% in cytotrophoblast cells isolated from human term placentas. Furthermore, we showed that sFlt-1 levels in HTR-8/SVneo cells, a cell line derived from first trimester trophoblast cells, decreased after exposure to very low concentrations of pravastatin (0.01, 0.1 µM). We also examined the effects of pravastatin on uterine spiral artery remodeling-related events and showed in wound healing and tube formation assays that low concentrations of pravastatin upregulated cell migration and invasion in HTR-8/SVneo cells. These results demonstrated that a low dose of pravastatin has in vitro effects that suggest a potential for anti-preeclamptic effects in vivo.

## Introduction

Preeclampsia (PE) is a hypertensive disorder characterized by proteinuria or organ failure with an onset after 20 weeks of gestation. PE affects approximately 3% to 8% of all pregnancies [1] and can be life-threatening to both the mother and child. Antihypertensive medications have only temporary effects. Administration of 75 to 150 mg of aspirin has been proposed as a prophylactic treatment, but it has limited efficacy [2–5]. To date, the only treatment for PE is delivery of the baby and placenta.

The placenta, and in particular placental trophoblast cells, plays a critical role in the pathogenesis of PE. During the early stages of pregnancy, cytotrophoblasts (CTBs) migrate from the tips of the villi and invade the endometrium. Invading CTBs, called extravillous trophoblasts (EVTs), are involved in vascular remodeling of the uterine spiral arteries. The Two-Stage Theory [6] suggests that a poor infiltration of EVTs into the uterine myometrium results spiral artery remodeling defects, resulting in hypoxic conditions in the placental circulation (first stage). Subsequently, serum soluble fms-like tyrosine kinase 1 (sFlt-1), an anti-angiogenic factor, is released into the maternal circulation, which causes maternal endothelial damage and results in hypertension and proteinuria (second stage). sFlt-1 levels are reported to be elevated in women with PE, and administration of sFlt-1 to rats causes a PE phenotype [7].

The statin pravastatin, a 3-hydroxy-3-methylglutaryl coenzyme A (HMG-CoA) reductase inhibitor, is used to treat hypercholesterolemia. Besides lowering cholesterol, statins have anti-inflammatory and antioxidant properties [8]. Because of these pleiotropic effects, statins are used as prophylactic agents for cardiovascular disorders and strokes [9–11]. Pravastatin is hydrophilic and less likely to cross the cellular lipid bilayer membrane, making it safer to use than lipophilic statins. In addition, pravastatin is classified as having low to moderate intensity in its cholesterol-lowering effects, which means that it has less effect on cholesterol levels during pregnancy. After publication of our finding that pravastatin can effectively prevent PE in vivo [12], several clinical studies have been conducted on the preventive and therapeutic effects of pravastatin in PE [13–18]. Some reported a reduction in the incidence of PE, improved perinatal prognosis, and decreased sFlt-1 levels; however, because of variations in the dosages and timing of pravastatin administration, the degree of anti-preeclamptic effects were inconsistent. Therefore, further studies are required to determine the optimal dose and timing of pravastatin administration. Before clinical trials are performed in pregnant women, in vivo and in vitro data need to be collected. Therefore, in this in vitro study, we examined the effects of low concentrations of pravastatin (0.01 to 10 µM) on PE-associated angiogenic factors, such as sFlt-1, placental growth factor (PlGF), and vascular endothelial growth factor (VEGF) (second stage) and on cell migration and invasion abilities related to placental spiral artery remodeling (first stage).

## Material and Methods

### Cell Culture

HTR-8/SVneo cells, a human first-trimester EVTs cell line, were kindly provided by Dr. Charles Graham (Queen’s University, Kingston, Ontario, Canada). The cells were maintained in RPMI-1640 (Wako Chemicals, Osaka, JAPAN) supplemented with 10% heat-inactivated fetal bovine serum (FBS) (Thermo Fisher Scientific, Waltham MA, USA) and a 100 × penicillin–streptomycin–amphotericin B suspension (Wako Chemicals) at 37 °C under normoxic conditions of 20% O_2_ and 5% CO_2_. They were plated at 5 × 10^4^/well on a 6-well plate, treated at 20% confluency with 0, 0.01, 0.1, 1, 5, or 10 µM pravastatin (Cayman Chemical, Ann Arbor, MI, USA), and incubated under hypoxic conditions for 48 h. For various experiments under hypoxic conditions, cells were incubated at 37 °C in a 9000EX (WAKENBTECH, Kyoto, Japan) at 93% N_2_, 2% O_2_, and 5% CO_2_. Conditioned media was collected for measurements of sFlt-1 and PlGF levels, and cell lysates were collected for RNA extraction. Pravastatin was dissolved in dimethyl sulfoxide (DMSO) as a stock solution, and the stock solution was added to the cell culture medium to obtain a final concentration of 0.1%. An equal volume of DMSO was added to the medium as a control.

### Isolation of Human CTBs

All experiments were performed with the approval of the Institutional Review Board of the Faculty of Medicine, University of Tokyo (IRB No. 11538). Normal term placentas were collected from 3 pregnant women who had undergone elective cesarean sections; informed consent was obtained from all 3 women. Human villous CTBs were isolated from the placentas according to protocols previously reported by our group [19]. Briefly, chorionic villous tissues were minced and digested in Hanks’ balanced salt solution containing trypsin (Thermo Fisher Scientific), DNase type 1, Dispase II (Sigma-Aldrich, St. Louis, MI, USA), CaCl_2_, and MgSO_4_. After filtering through a 100-μm nylon filter, CTBs were selected by Percoll (GE Healthcare, Chicago, IL, USA.) density gradient centrifugation. The collected CTBs were incubated with anti-HLA-ABC antibodies (Novus Biologicals, Littleton, CO, USA). HLA-ABC-negative cells were filtered and isolated with a Mini MACSTM separator (Miltenyi Biotec, Bergisch Gladbach, Germany) containing anti-mouse IgG antibody microbeads. Purified CTBs were diluted in IMDM Glutamax (Thermo Fisher Scientific), supplemented with 10% FBS and 100 × penicillin–streptomycin–amphotericin B suspension, and plated at a density of 1 × 10^6^ cells/mL in 6-well plates coated with collagen type 1 (IWAKI, Shizuoka, Japan). The cells were treated with 0 -, 0.01 -, 0.1 -, 1 -, 5 -, and 10 μM pravastatin and incubated under hypoxic conditions for 72 to 96 h. Each experiment used CTBs isolated from the placenta of 1 woman, and CTBs from the 3 women were not mixed. Depending on the number of cells purified, 1 to 3 experiments per placenta were performed.

### Cell Viability Assays

After culturing HTR-8/SVneo cells (5 × 10^3^ cells/well) in a 24-well plate for 24 h, we treated the cells with 0 -, 0.01 -, 0.1-, 1 -, 5 -, or 10 μM pravastatin under hypoxic conditions. After 48 h, Cell Counting Kit-8 reagent (DOJINDO, Kumamoto, Japan) was added to the media at a 1/10 concentration, wells were incubated for an hour, and the optical density was measured at 450 nm.

### Enzyme-Linked Immunosorbent Assays (ELISAs)

The human sFlt-1 concentration in HTR-8/SVneo cell culture supernatants was measured with a human VEGF R1/Flt-1 ELISA Quantikine kit (R&D Systems, Minneapolis, MN, USA) according to the manufacturer’s guidelines. The minimum detection limit of these assays was 8.46 pg/mL for sFlt-1. The concentration of human sFlt-1 in CTBs was determined with a human VEGF R1/Flt-1 DuoSet (R&D Systems). The concentrations of PlGF and VEGF in both cell types were measured with human PlGF DuoSets and human VEGF DuoSets (R&D Systems). Data are presented as relative change compared to control, with control as 100%.

### RNA Extraction and Quantitative Real-Time Polymerase Chain Reaction (PCR)

Total RNA was extracted from cells with a Blood/Cultured Cell Total RNA Mini Kit (FAVORGEN, Ping-Tung, Taiwan) according to the manufacturer’s instructions. Total RNA was reverse-transcribed into cDNA by using ReverTra Ace® qPCR RT Master Mix (TOYOBO, Osaka, Japan). Quantitative real-time PCR was performed with SYBR Green PCR Master Mix (Roche Diagnostics KK, Tokyo, Japan) and the QuantStudio 1 Real-Time PCR System (Applied Biosystems, Foster City, CA, USA). The expression levels of the target genes were normalized to β actin (internal control), and relative fold changes were calculated using the ΔΔCT method. The sequences of the oligonucleotides used as primers were as follows: sFlt-1 forward 5’- CTCCTGCGAAACCTCAGTG -3’, reverse 5’- GACGATGGTGACGTTGATGT -3’, PlGF forward 5’-GTTCAGCCCATCCTGTGTCT-3’, reverse 5’-AACGTGCTGAGAGAACGTCA-3’, VEGF forward 5’-TGCAGATTATGCGGATCAAACC-3’, reverse 5’-TGCATTCACATTTGTTGTGCTGTAG-3’, MMP (matrix metalloproteinase) 1 forward 5’-ACATGAGTCTTTGCCGGAGG-3’, reverse 5’-ATCCCTTGCCTATCCAGGGT-3’, MMP2 forward 5’-GCTGGCTGCCTTAGAACCTTTC-3’, reverse 5’- GAACCATCACTATGTGGGCTGAGA-3’, MMP9 forward 5’- GCCACTACTGTGCCTTTGAGTC-3’, reverse 5’- CCCTCAGAGAATCGCCAGTACT-3’, β actin forward 5’-CATGTACGTTGCTATCCAGGC-3’, reverse 5’-CTCCTTAATGTCACGCACGAT-3’.

### Wound Healing Assay

After HTR-8/SVneo cells reached subconfluence in the 6-well plates, they were incubated and starved in serum-free medium for 6 h. Subsequently, the cell monolayers were scratched with a sterile P200 pipette tip to create a wound, and the conditioned media were replaced with treatment media containing different concentrations of pravastatin (0, 0.01, 0.1, 1, 5, or 10 µM). Inverted phase-contrast microscopy images were captured immediately after scratching and after incubation under hypoxic conditions for 16 h. Non-populated areas were quantified by ImageJ software (National Institutes of Health, Bethesda, MD, USA) and subtracted to obtain the wound closure rate, which was calculated from the equation {(T0-T16)/T0} × 100 (%), where T0 is the non-populated area immediately after scratching and T16 is that after 16 h. Data are presented as relative change compared to control, with control as 100%.

### Tube Formation Assay

The surfaces of the 96-well plates were coated with 30 µL of growth factor-reduced Matrigel (BD Biosciences, Bedford, MA, USA) according to the manufacturer’s instructions. After the HTR-8/SVneo cells were incubated in serum-free medium for 6 h, they were harvested into Matrigel-coated wells (1 × 10^4^ cells/well) and treated with 0 -, 0.01 -, 0.1 -, 1, 5 -, or 10 µM pravastatin without FBS under hypoxic conditions for 16 h. Microvascular tube formation was observed with an inverted phase-contrast microscope at 4 × magnification. The total tube length was quantified with ImageJ imaging software (National Institutes of Health). Data are presented as relative change compared to control, with control as 100%.

### Statistical Analyses

Statistical analyses were performed with JMP® Pro 17 software (SAS Institute, Cary, NC, USA). All data are expressed as the mean ± SD. Steel’s test was used to analyze non-parametric data. Statistical significance was set at a P-value of less than 0.05.

## Results

### Low Concentrations of Pravastatin Do not Reduce Cell Viability in CTBs

**Fig. 1:**
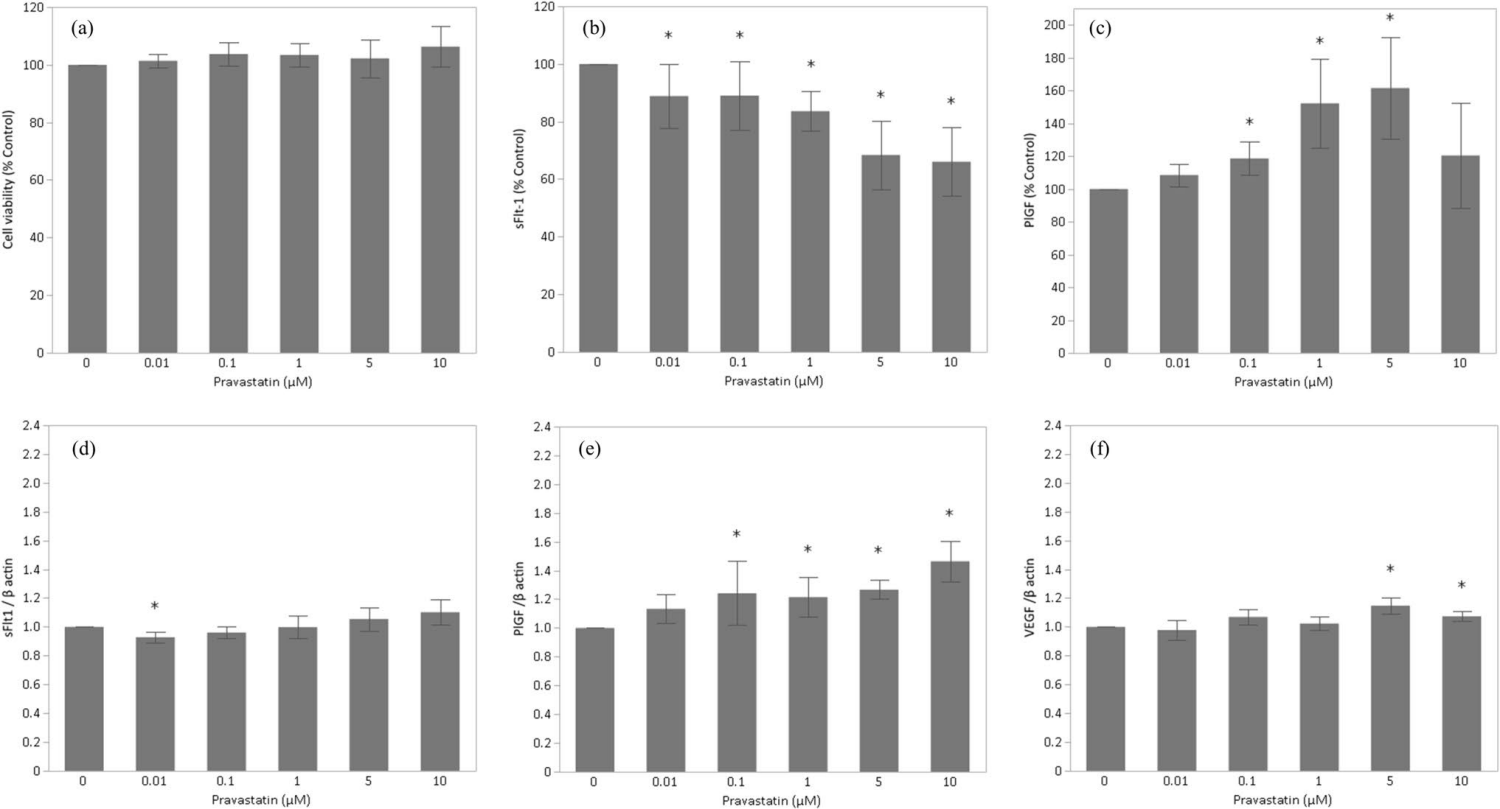
Pravastatin effects on CTBs. **a** Cell viability (*n* = 6), **b** sFlt-1 protein (*n* = 6), **c** PlGF protein (*n* = 6), **d** sFlt-1 mRNA expression (*n* = 6), **e** PlGF mRNA expression (*n* = 6), and **f** VEGF mRNA expression (*n* = 6). Steel’s test was used to determine statistical significance (*p < 0.05)

The maternal characteristics of the isolated CTBs refer to Table 1. To determine whether pravastatin is toxic to CTBs, cell viability was assessed after the addition of pravastatin. Pravastatin administered at any concentration did not affect CTB viability (Fig. 1a).

**Table 1:** Maternal characteristics of isolated CTBs

	Case1	Case2	Case3
Maternal age	31	38	29
Gestational age of delivery	38w3d	38w0d	38w1d
Indications for cesarean section (CS)	Previous CS	Previous CS	Previous CS

### sFlt-1 and PlGF Levels in CTBs Are Altered by Low Concentrations of Pravastatin

ELISA assays were performed to determine the concentrations of the anti-angiogenic factor sFlt-1 and the pro-angiogenic factors PlGF and VEGF in cell culture supernatants. After treatment with pravastatin, sFlt-1 levels in CTBs decreased in a concentration-dependent manner, up to 34%, whereas PlGF levels increased from 0.01 to 5 µM, up to 62% (Fig. 1b-c); VEGF protein was not detected following treatment with pravastatin at any concentration (data not shown).

### sFlt-1, PlGF, and VEGF mRNA Expression Levels of CTBs Are Affected by Low Concentrations of Pravastatin

Treatment with pravastatin led to a decrease in sFlt-1 mRNA at 0.01 µM and then progressively increased, an increase in PlGF mRNA in a concentration-dependent manner, and an increase in VEGF mRNA at 5 and 10 µM (Fig. 1d-f).

### Low Concentrations of Pravastatin Do not Reduce Cell Viability in HTR-8/SVneo

**Fig. 2:**
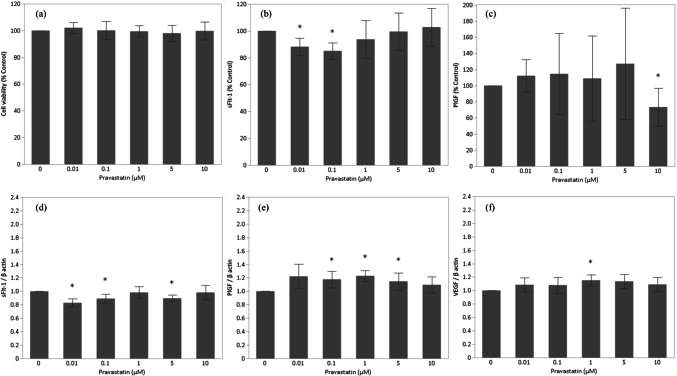
Pravastatin effects on HTR-8/SVneo cells. **a** Cell viability (*n* = 10), **b** sFlt-1 protein (*n* = 6), **c** PlGF protein (*n* = 5–6), **d** sFlt-1 mRNA expression (*n* = 6), **e** PlGF mRNA expression (*n* = 6), and **f** VEGF mRNA expression (*n* = 6)

The viability of EVTs treated with low concentrations of pravastatin did not differ from that of control cells (Fig. 2a).

### sFlt-1 Levels in HTR-8/SVneo Are Decreased by Low Concentrations of Pravastatin

The lowest concentrations of pravastatin (0.01 and 0.1 µM) decreased sFlt-1 levels but did not affect PlGF levels (Fig. 2b-c). In contrast, 10 µM pravastatin decreased PlGF levels but did not affect sFlt-1 levels. VEGF protein was not detected (data not shown).

### PlGF and VEGF but not sFlt-1 mRNA Expression Levels of HTR-8/SVneo Are Induced by Low Concentrations of Pravastatin

sFlt-1 expression decreased in the presence of 0.01-, 0.1-, and 5 µM pravastatin (Fig. 2d). In contrast, PlGF expression increased after treatment with 0.1- to 5 µM pravastatin (Fig. 2e). VEGF mRNA expression was also induced by the addition of 1 µM pravastatin (Fig. 2f).

### Low Concentrations of Pravastatin Increase HTR-8/SVneo Migration

**Fig. 3:**
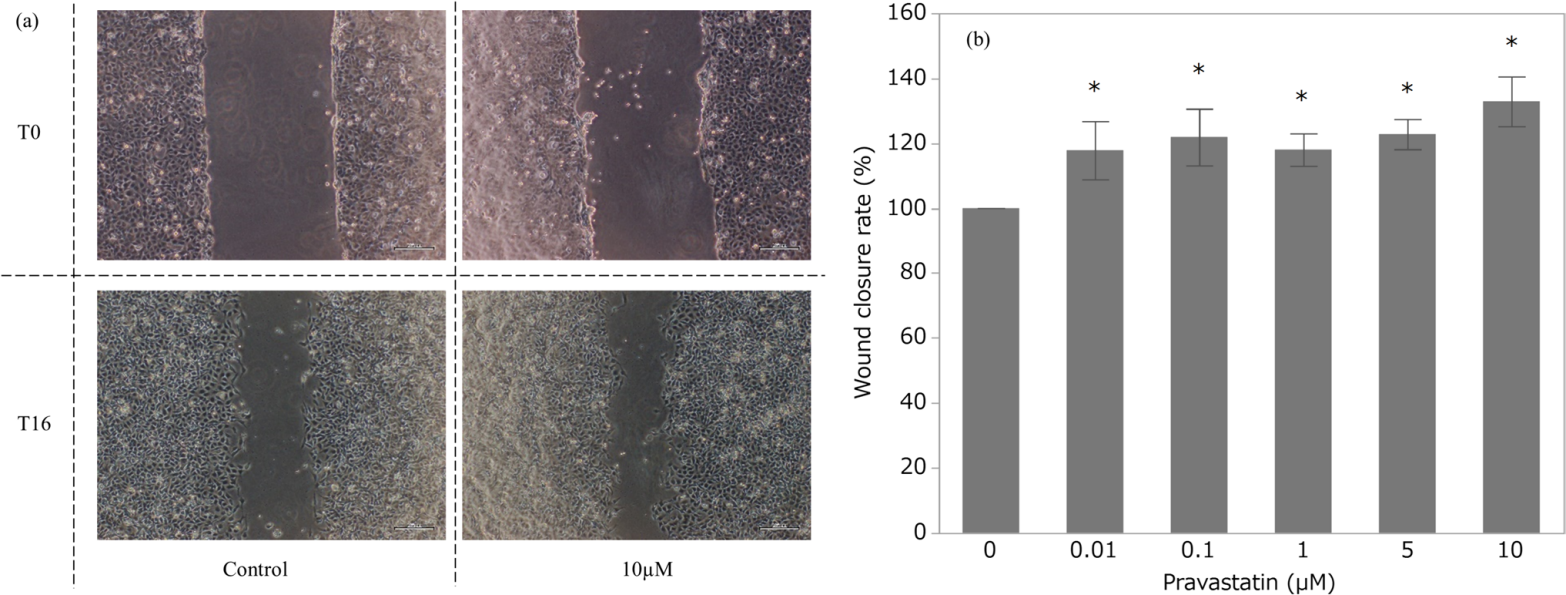
Wound healing assay. **a** Inverted phase-contrast microscopy images, and **b** wound closure rate (*n* = 12)

The wound closure rate was higher after exposure to any pravastatin concentration, with increases of up to 32%. (Fig. 3a-b).

### Low Concentrations of Pravastatin Induce HTR-8/SVneo Angiogenesis

**Fig. 4:**
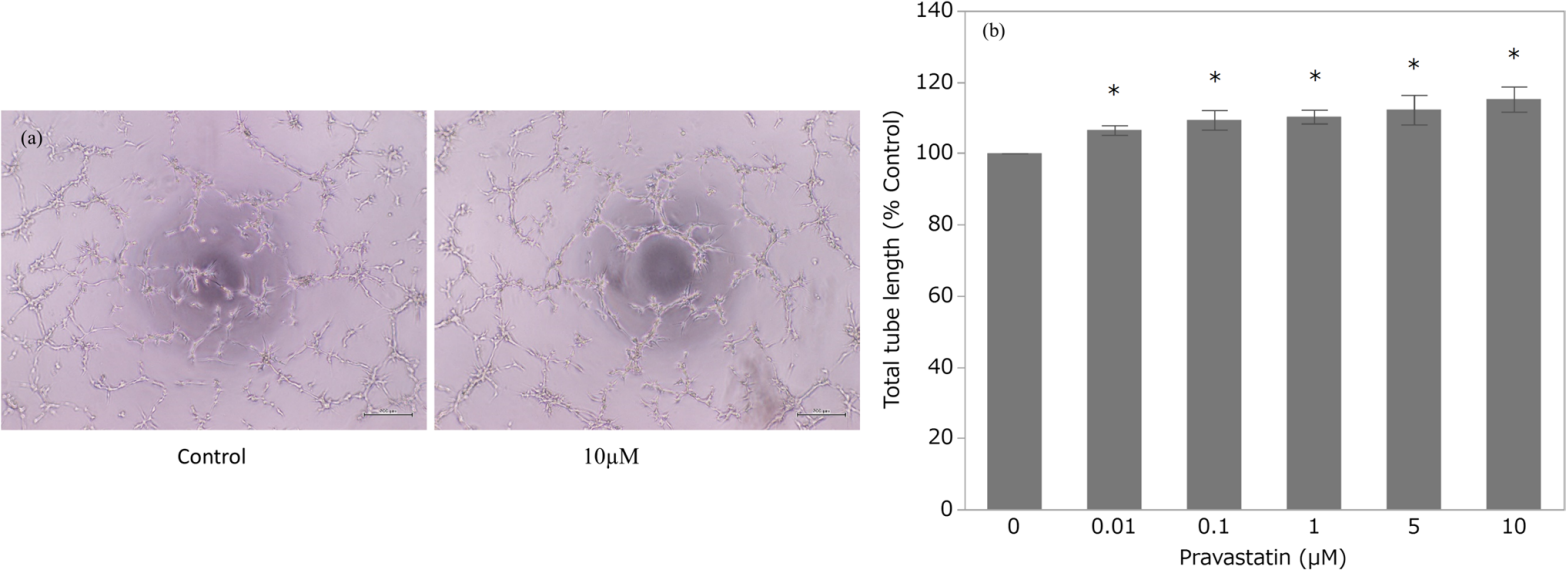
Tube formation assay. **a** Inverted phase-contrast microscopy images, and **b** total tube length (*n* = 12)

The total tube length of the tubular networks increased after exposure to any pravastatin concentration, with increases of up to 15% (Fig. 4a-b).

### Low Concentrations of Pravastatin Enhance MMPs mRNA Expression in CTBs and HTR-8/SVneo

**Fig. 5:**
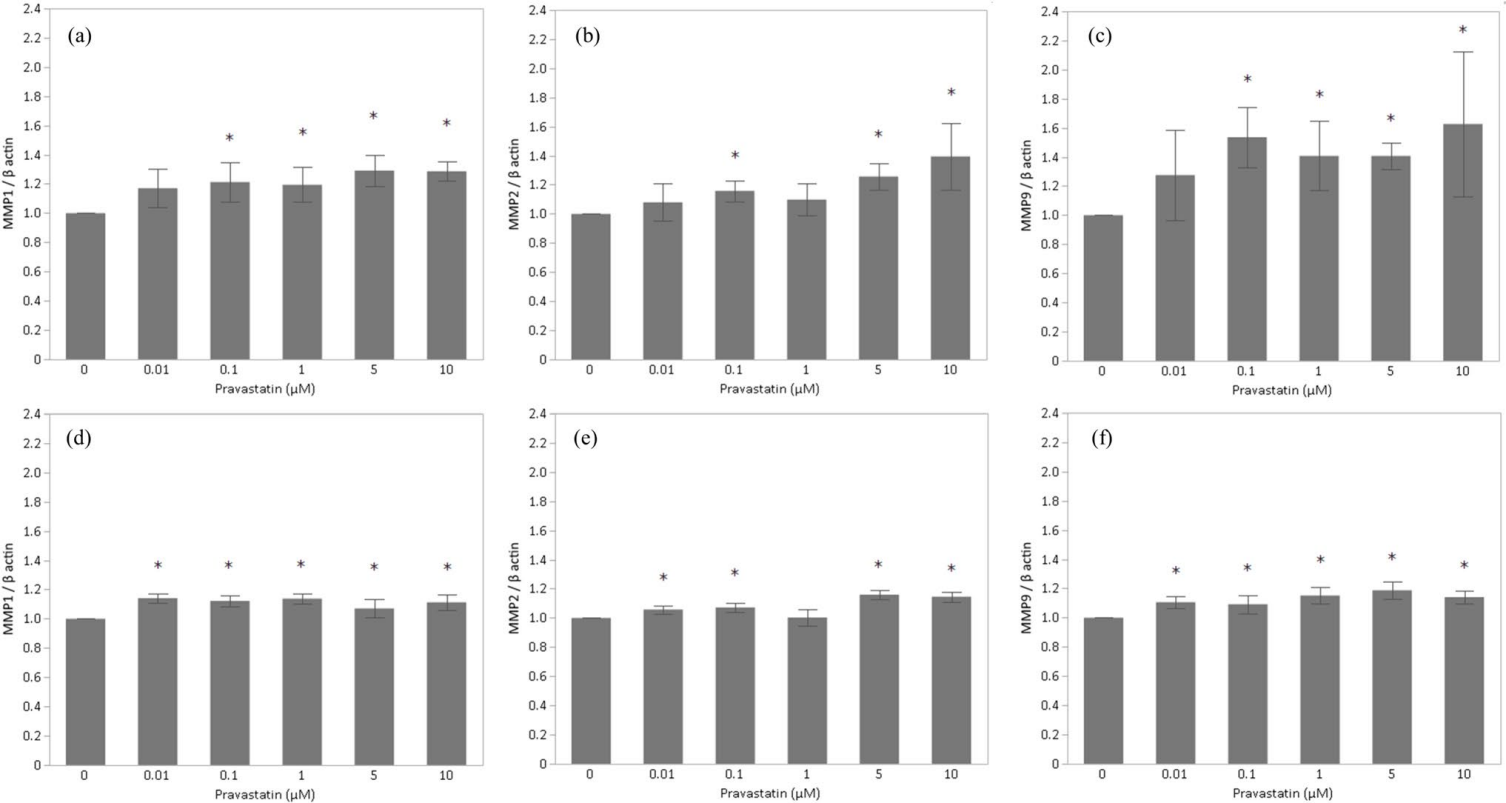
Pravastatin effects on MMPs mRNA expression. **a** MMP1 expression of CTBs (*n* = 6), **b** MMP2 expression of CTBs (*n* = 6), **c** MMP9 expression of CTBs (*n* = 6), **d** MMP1 expression of HTR-8/SVneo cells (*n* = 8), **e** MMP2 expression of HTR-8/SVneo cells (*n* = 8), and **f** MMP9 expression of HTR-8/SVneo cells (*n* = 8)

To investigate the mechanisms underlying cell migration and angiogenesis, we examined MMPs mRNA expression levels in CTBs and HTR-8/SVneo cells. MMPs have been implicated in extracellular matrix remodeling and trophoblast invasion of the spiral arteries during pregnancy. Furthermore, expression of MMP1, MMP2, and MMP9 is known to be lower in animal models of PE and in the placenta of patients with PE [20–22]. We found that MMP1 and MMP9 mRNA expression in CTBs was upregulated compared with controls at pravastatin concentrations ranging from 0.1 to 10 µM (Fig. 5a, c). MMP2 mRNA expression was upregulated after treatment with 0.1-, 5-, and 10 µM pravastatin (Fig. 5b). In HTR-8/SVneo cells, MMP1 and MMP9 mRNA expression increased after treatment at all pravastatin concentrations (Fig. 5d, f). MMP2 mRNA expression increased with 0.01-, 0.1-, 5-, and 10 µM pravastatin (Fig. 5e).

## Discussion

In the past decade, several clinical studies have evaluated the potential use of pravastatin for PE [13–18]. However, to date, the timing and dosage of pravastatin administration have not been established, and the search for the optimal administration regimen remains a critical issue. In vitro studies reported that statins have concentration-dependent biphasic proliferative effects, i.e., angiogenesis is induced at low concentrations of statins and inhibited at high concentrations [23, 24]. Therefore, we hypothesized that low-dose pravastatin would improve placentation during early pregnancy and decrease sFlt-1 levels in later pregnancy.

In this study, we used HTR-8/SVneo cells, a cell line of EVTs, to reflect the placenta in early pregnancy and CTBs to reflect the placenta in late pregnancy. CTBs have been widely used to evaluate the effects of various drugs, such as chemotherapeutic agents for perinatal complications and insulin and metformin for the placenta [25, 26]. HTR-8/SVneo cells are also widely used in PE research [27].

We also focused on hypoxic conditions. Because of the placenta has an insufficient blood supply, the placenta in patients with PE is known to be under more hypoxic conditions than in patients with a normal pregnancy [28]. Hypoxic stimulation has been widely used in vitro to reproduce placental conditions of PE more accurately [20, 29, 30]. In this study, we used HTR-8/SVneo cells and CTBs with hypoxia as ‘PE model cells’ to examine the effect of low concentrations of pravastatin on both the first and second stages of PE as defined by the Two-Stage Theory. We found that low concentrations of pravastatin decreased sFlt-1 protein levels of CTBs and HTR-8/SVneo cells suggesting that pravastatin may ameliorate the second stage of PE development. Based on these findings, low-dose pravastatin may have the potential to prevent PE.

Serum PlGF levels are also associated with PE pathology in that PlGF levels are significantly lower in women with PE than in pregnant women without PE [7, 31]. We previously reported that pravastatin administration induces PlGF and improves symptoms in a PE mouse model [12]. In the present study, low concentrations of pravastatin had a tendency to increase PlGF protein in CTBs in addition to its sFlt-1–lowering effect. The reason why the VEGF protein was not detected in this study is speculated to be that the small amount of VEGF secreted into the supernatant was neutralized by binding to sFlt-1 [7].

In this study, we also showed that low concentrations of pravastatin improved both migration and angiogenesis of HTR-8/SVneo and upregulated MMP mRNA, suggesting that pravastatin may ameliorate the first stage of PE development. Migration and tube formation assays are widely used to evaluate angiogenesis [32]. The results of our migration and tube formation assays are consistent with our previous study in mice, which revealed that placental angiogenesis recovered in PE mice treated with low-dose pravastatin [12]. Our results with MMPs also suggest an improvement in the migratory and invasive capacity of trophoblasts, indicating that pravastatin ameliorates insufficiencies in trophoblast invasion in the maternal myometrium and defects in spiral artery remodeling. Taken together, the findings are consistent with the results of various clinical trials showing that pravastatin administration at early gestational weeks may have a preventive effect against PE [15, 16, 18].

As for the toxicity of pravastatin in placental cells, Brownfoot et al. [33] reported that cell viability was maintained when placental cells were treated with 2,000 µM pravastatin, which is a much higher concentration than was used in our study. The low concentrations of pravastatin used in our study did not affect the viability of either CTBs or HTR-8/SVneo cells.

One of the limitations of our study is that we examined the anti-PE effect only in vitro. Because pravastatin is already prescribed for pregnant women at high risk for PE, we examined the effects of pravastatin on cells derived from human placentas. Another key limitation of this study is that we collected CTBs from normal term placentas, i.e., after the third trimester of pregnancy, we did so because early pregnancy placentas do not contain a sufficient number of cells for our protocol. To complement this approach, we used a human EVT first-trimester placentas cell line, HTR-8/SVneo, to test the effects of pravastatin in early pregnancy.

Although low concentrations of pravastatin decreased sFlt-1 protein levels in both CTBs and HTR-8/SVneo cells, the pattern of reduction was not the same. This difference may be partly due the different timing of cell collection: HTR-8/SVneo cells were derived from first trimester, but sFlt-1 starts to be released predominantly in the second trimester. Moreover, the difference may also arise from differences between EVTs and CTBs and between primary cells and cell lines. Nevertheless, in both cell types sFlt-1 protein levels were decreased after treatment with low concentrations of pravastatin.

In addition to angiogenic balance, PE is also associated with increased oxidative stress, an imbalance between pro-inflammatory and anti-inflammatory cytokines, and systemic endothelial dysfunction. Statins are reported to activate the heme oxygenase-1 (HO-1)/carbon monoxide pathway, which has antioxidant, anti-inflammatory, and vasoprotective properties, resulting in suppression the production of sFlt-1 [34, 35]. In a study using human placenta-derived mesenchymal stem cell, HO-1 not only increased the expressions of PlGF and VEGF and decreased those of sFlt-1, but also improved the migration and tube formation capacities, suggesting improved placental angiogenesis [36]. Further research is needed to elucidate the effects of low concentrations of pravastatin on these pathogeneses.

Although statins are contraindicated during pregnancy in several countries, a large-scale cohort study [37] and a meta-analysis of cohort studies on statin exposure during pregnancy [38] found that statin exposure was not significantly associated with the incidence of congenital anomalies. Subsequently, various clinical studies with pravastatin were conducted. Furthermore, pravastatin administration during pregnancy did not worsen placental function [15] and did not adversely affect the long-term neurological prognosis of the child [39]. The results of meta-analyses of anti-PE effects were controversial, which could be attributed to the various initiation timings and doses of pravastatin.

## Conclusion

In the in vitro present study, we used PE model cells and demonstrated that low concentrations of pravastatin reduced sFlt-1 levels. Moreover, low concentrations of pravastatin improved cell migration and angiogenesis, suggesting better placentation in early pregnancy. Our results indicate that administration of low-dose pravastatin in early pregnancy may improve both first- and second-stage pathologies and prevent progression to PE, and they warrant further evaluation in future clinical studies. A randomized controlled trial in pregnant women at high risk for PE is required to confirm the effectiveness and safety of low-dose pravastatin in this population.

## Data Availability

The data are available from the corresponding author on reasonable request.
